# Cholecystectomy for Complicated Gallbladder and Common Biliary Duct Stones: Current Surgical Management

**DOI:** 10.3389/fsurg.2020.00042

**Published:** 2020-07-21

**Authors:** Yanna Argiriov, Melanie Dani, Christos Tsironis, Louis J. Koizia

**Affiliations:** ^1^Cutrale Perioperative and Ageing Research Group, Department of Bioengineering, Imperial College London, London, United Kingdom; ^2^Department of Surgery, Imperial College Healthcare NHS Trust, London, United Kingdom

**Keywords:** cholecystectomy, early cholecystectomy, delayed cholecystectomy, gallstones, biliary disease

## Abstract

Gallstone disease accounts for the vast majority of acute surgical admissions in the UK, with a major treatment being cholecystectomy. Practice varies significantly as to whether surgery is performed during the acute symptomatic phase, or after a period of recovery. Differences in practice relate to operative factors, patient factors, surgeon factors and hospital and trust wide policies. In this review we summarize recent evidence on management of gallstone disease, particularly with respect to whether cholecystectomy should occur during index presentation or following recovery. We highlight morbidity and mortality studies, cost, and patient reported outcomes. We speculate on barriers to change in service delivery. Finally, we propose potential solutions to optimize care.

## Introduction and Objectives

Gallstone-related disease was the commonest cause of hospital admissions in the developed world at the beginning of the 21st Century (1, 2). In developed nations, gallstones affect 10–20% of adults (3–5), of whom 80% are asymptomatic (5, 6). The remainder can present with anything from biliary colic, cholecystitis, cholangitis, choledocholithiasis (i.e., common bile duct [CBD] stones) and gallstone pancreatitis (7–11), to rarer severe variants such as Mirizzi's syndrome, gangrenous, haemorrhagic, or emphysematous cholecystitis. The latter group tend to have non-specific clinical, serological and radiological findings which can make diagnosis difficult (12–25).

Tokyo Guidelines 2018 (TG18) acknowledge that gallstone disease can present diversely and non-traditionally and outline various workup and management strategies according to patients' comorbidities, presentation, biomarkers, diagnosis and disease severity (26).

Under TG18, three key criteria must be present to diagnose acute cholangitis and acute cholecystitis, respectively (27). For a definite acute cholangitis diagnosis there must be: (1) Leucocytosis >10 × 10^9^/L and CRP>10 mg/L, and/or fevers/chills, plus (2) Consistent imaging findings (described below), plus (3) signs of cholestasis. This can be jaundice/ bilirubin >20 mg/L or deranged liver function tests (LFTs): i.e., alkaline phosphatase (ALP), γ-glutamyl transpeptidase (GGT), alanine aminotransferase (ALT), and aspartate aminotransferase (ALT) at 1.5x their upper limit of normal (28). GGT in particular has high positive and negative predictive values, with 10x higher probability of CBD stones when GGT>90 units/L (29).

For acute cholecystitis, points (1) and (2) are as above (with absence of “chills” in point 1), but the third criterion differs in that jaundice and deranged LFTs are not part of an acute cholecystitis diagnosis. Instead, there must be a mass, pain or tenderness in the right upper quadrant, or a positive Murphy's sign (30).

Transabdominal ultrasound is the gold-standard imaging for acute cholecystitis and detects over 80% of cases (31–35). In acute uncomplicated cholecystitis, ultrasound may show gallstones, a positive sonographic Murphy's sign, thickened gallbladder wall, pericholecystic fluid, “sludge” in the gallbladder, gallbladder distension, and hyperaemia of the gallbladder wall on Doppler (30, 36–38). However, it is limited in detecting severe variants such as gangrenous or emphysematous cholecystitis or intra-abdominal abscess, where CT is more appropriate (14, 24, 30, 37). Cholescintigraphy has shown to be diagnostically superior to ultrasound, with smaller error margins (39, 40), but is limited in practice as it can take several hours, irradiates the patient and cannot look beyond the hepatobiliary tract (41). Ultrasound therefore remains recommended by the National Institute for Health and Care Excellence (NICE) (42).

In choledocholithiasis, abdominal ultrasound may detect stones or dilation of the CBD bile duct (28, 33), but not consistently. Magnetic Resonance Cholangiopancreatography (MRCP) is more sensitive for CBD abnormalities, and can be used if ultrasound is inconclusive (28). The Association of Upper Gastrointestinal Surgery of Great Britain and Ireland (AUGIS) suggest MRCP only for high-risk patients: i.e., those with CBD >10 mm on ultrasound with abnormal LFTs and bilirubin. Even then, cholecystectomy with intra-operative cholangiography (IOC) is preferable (43). Other guidelines also propose MRCP for intermediate-risk patients (although do acknowledge IOC as an alternative) (44–46).

Once identified, CBD stones should be removed within 72 h if the patient is jaundiced, or within 24 h for acute cholangitis or pancreatitis (47). This should be by pre-operative Endoscopic Retrograde Cholangiopancreatography (ERCP) or intra-operative laparoscopic bile duct exploration plus cholecystectomy (43).

Indeed, laparoscopic cholecystectomy is the gold-standard treatment for symptomatic gallstones (45, 48–51). For those with acute cholecystitis, NICE recommends laparoscopic cholecystectomy within 1 week of presentation; within 72 h is the ideal (42, 52–54). Almost 70,000 cholecystectomies are performed each year in the UK (costing more than £110 million) (55). Despite recommendations, only 15% of these are done early (i.e., on index admission) (56, 57), with patients instead being discharged and operated on electively.

This article will discuss early vs. delayed laparoscopic cholecystectomy, discuss reasons for operating delays, and propose how to mitigate these delays in the UK health service.

## Why Early Cholecystectomy is Important

### Safety

In the 1990's, studies suggested early cholecystectomy had higher conversion rates, longer operation times and increased risk of complications (58–61). However, countless studies since proved early procedures are safe (62, 63), some of which we will now discuss.

In 2015, Cao et al. published a meta-analysis of 14 randomized control trials involving 1,608 patients showing that morbidity was double in patients undergoing delayed cholecystectomy (30 vs. 15%), with higher rates of wound infection. Conversion rates were the same regardless of procedure timing (64), although some studies have found conversion rates significantly lower in early groups (52, 65–68).

In older patients, morbidity in delayed procedures is higher still. Cull et al. (69) reviewed 265 cholecystitis patients over 65 years-of-age, and found those undergoing cholecystectomy over 7 days from symptom-onset were four times more likely to develop recurrent biliary tract infection prior to surgery compared to those that had cholecystectomy within 7 days. Two percentage of the delayed cholecystectomy group died, whereas there were no deaths in the early group, although this was not statistically significant (69).

A previous concern of early laparoscopic cholecystectomy was heightened risk of bile duct injury, due to inflammatory adhesions to adjacent structures (70). However, repeated attacks of cholecystitis (as can occur in elective patients) can also result in gallbladder adherence to surrounding structures, making surgery equally problematic (62, 71–73).

A large Canadian retrospective cohort study of 22,202 acute cholecystitis patients found early cholecystectomy in fact carries significantly lower probability of major bile duct injury (relative risk 0.53) with no statistically significant difference in mortality or conversion rates (74). Recently, even larger studies have supported this finding (52, 75).

A meta-analysis of 451 patients by Gurusamy et al. (76) found 17.5% of interval cholecystectomy patients re-presented and required emergency surgery before their planned date. Of these patients, 45% required conversion to an open operation, more than double the originally-stated interval group conversion rate. This suggests that conversion and morbidity may be significantly higher in delayed cholecystectomy groups when those requiring emergency interventions are considered. It has even been suggested that delaying cholecystectomy increases risk of severe variants such as gangrenous cholecystitis (77, 78).

Early cholecystectomy is also beneficial in acute cholangitis. Discolo et al. (79) compared early and late cholecystectomy in acute cholangitis and found those undergoing delayed procedures were more likely to have post-operative complications, and/or recurrent cholangitis before their surgery. Differences in intra-operative complication rates were negligible. Similarly, delaying cholecystectomy for gallstone pancreatitis patients has also been linked to recurrence and increased complications (80).

### Readmissions and Length of Stay

Increased morbidity in delayed cholecystectomy, as discussed above, is in part due to emergency gallstone-related readmissions whilst awaiting surgery (81), rates of which can be as high as 20–29% (82–86). For those awaiting cholecystectomy for more than 20 weeks, readmission rates increase threefold (87).

Total length of stay is also longer when cholecystectomy is delayed. Papi et al. (62) showed that delayed cholecystectomy patients stayed in hospital an extra 8.2 days on average, compared to the early group.

Some studies show the difference to be smaller (around 2–4 days) (63, 74, 85, 88–91), but all studies looking at length of stay found it shorter for early procedures (52, 64, 76, 84, 92–94), even when comparing open cholecystectomy approaches (95).

### Patient Reported Outcomes

In elective patients, waiting for surgery leads to a prolonged period of impaired health with psycho-social implications for patients.

Oudhoff et al. (96) found patients awaiting cholecystectomy exhibit signs of lethargy and anxiety which are positively correlated to symptomatology, complications and readmission, and improve once the procedure is done. Women awaiting cholecystectomy had higher social dysfunction than women with breast cancer awaiting biopsy (96).

A further qualitative study by Lindseth and Denny (97) featured quotes from patients awaiting cholecystectomy electively; some describing the pain as “*excruciating.”* Patients unanimously described inability “*to enjoy eating or follow their usual dietary habits”* and some “*had significant weight loss… afraid to eat for fear of the pain.”* Sleep disturbance pre-operatively was also common (97).

### Cost

Repeated gallstone-disease flares increase spend due to repeated admissions and longer average bed stays (62, 84, 98). A UK study by Jones et al. (88) showed that inpatient bed costs were the second-largest cholecystectomy spend after the operation itself and could reach up to £2849 per patient. By reducing length of stay, early cholecystectomy could result in significant cost-savings for hospitals.

Various UK-based literature has outlined cost-effectiveness of early procedures for acute cholecystitis. Wilson et al. (99) found early procedures were overall £820 cheaper per patient and could save the NHS £821,540 for every thousand patients treated, totalling £8.5 million savings per annum. Early cholecystectomy patients also scored better on Quality-Adjusted Life Year (QALY) criteria (early: £2,574,457 per 1,000 patients, QALYs 876.48. Delayed: £3,395,997 per 1,000 patients, QALYs 825.05) with a hospital stay 4.12 days shorter (99).

More recent UK economic evaluation by Kerwat et al. (100) found early laparoscopy was on average £645 cheaper per patient and could save the NHS £27million per annum if implemented as standard practice. The discrepancy between estimated NHS savings between studies is likely due to differences in NHS reference costings when the studies were published (2018 vs. 2010).

Morris et al. (101) looked at cost in gallstone pancreatitis patients and found the longer the procedure was postponed, the higher the cost. And even if index admission procedures are done after 72 h, they are still cheaper than discharging patients for interval cholecystectomy (101).

A costing statement by NICE also states that early cholecystectomy could save money from reduced A&E visits and fewer prescriptions for analgesia and antibiotics (55). Moreover, NHS tariff prices are considerably higher for emergency laparoscopic cholecystectomy patients than for delayed ones. According to NHS planning prices 2019/20, trusts are paid £3606 for a “hot” laparoscopic cholecystectomy, vs. £1855 for an elective one. In complex and comorbid patients, emergency tariff payment can be as high as £6333 (102). So not only do early procedures save money in terms of bed stay and treatment, they also generate more tariff income.

When considering cost, lost workdays should also be taken into account as this has financial impact on both patients and society as a whole. Gallbladder disease is the most expensive chronic gastrointestinal disorder with regards to lost workdays (103) and socioeconomic cost (104). Early cholecystectomy can reduce lost workdays by up to 11 days (71, 89, 105).

## Issues With Early Cholecystectomy

Early cholecystectomy is not without disadvantages. These will now be discussed.

A meta-analysis of 375 patients by Siddiqui et al. (93) found operating time was 2–45 min longer in early cholecystectomy for acute cholecystitis. Gul et al. (105) found mean operating time 18 min longer in the early cholecystectomy group and Wu et al. (89) found it 10 min longer. However, multiple other studies have shown negligible or no differences in operation time (84, 85, 92, 98, 106). A recent study published in 2019 even showed early procedures to have *reduced* operation time (107). Differences in early and delayed operation times are also minimal in acute cholangitis (97 vs. 89 min, respectively) (79). Complication and conversion rates were similar regardless of cholecystectomy length.

Although not all studies commented on blood loss, those that did reported early laparoscopic cholecystectomies to have mean extra blood loss of 13–120 ml in early procedures (105, 106, 108–110). This said, other literature does also exist citing differences in blood loss to be insignificant, or even reduced, in early procedures (111, 112).

Importantly, it should be noted that early cholecystectomy is not appropriate for all patients, and this is acknowledged in TG18 (113). In those with high comorbidity scores, poor performance statuses, jaundice, cranial neuropathy, respiratory dysfunction, or organ failure that is not rapidly reversible, percutaneous transhepatic gallbladder drainage (PTGD) followed by delayed cholecystectomy at 6 weeks is preferable with better morbidity outcomes (113–115). In these cases, we would not advocate early procedures.

In 2018, Blythe et al. reported that in the “real-world” early cholecystectomy has *increased* morbidity, mortality and complication rates compared to delayed (116). However, a key issue with the study was that those assigned to the “early” group were already significantly more unwell, had more complex cholecystitis variants (including abscess, empyema, ulceration, necrosis) and higher American Society of Anesthesiologists (ASA) scores: 6.5% of the early group had a pre-operative ASA score of IV, compared to 0% in the delayed group. Therefore, the results do not accurately reflect daily practice.

## Reasons for Delays

In 2004, Cameron et al. found only 11% of surveyed general surgical consultants performed early cholecystectomy for acute cholecystitis. Limiting factors were unavailability of experienced surgeons, limited theater space, and awaiting radiological investigations (117).

A larger survey in 2010 showed that 58% of surgeons performed index cholecystectomy for gallstone pancreatitis, but only 20% did for acute cholecystitis. Upper gastrointestinal and hepato-pancreato-biliary surgeons had much higher rates of index admission cholecystectomies compared to general surgeons (37 vs. 13%). Procedures were mostly performed in emergency theaters, followed by the earliest available elective list. Limiting factors to early operation were theater availability and imaging delays. The authors suggested that more early cholecystectomies could be done if performed by upper gastro-intestinal or hepato-pancreato-biliary surgeons, or those performing laparoscopic cholecystectomy regularly. They also proposed a 12-h waitlist theater that runs separately from CEPOD (118).

In addition to the above, AUGIS also identifies surgeon apprehension, uncertain use of bile duct imaging (e.g., MRCP) and low rates of operative cholangiography and laparoscopic bile duct clearance in the UK as causes for delays (43).

## Proposed Ways of Reducing Time to Cholecystectomy

There are several ways we could reduce time to cholecystectomy.

Firstly, is by curtailing unnecessary MRCP use. One way this could be achieved is through stricter adherence to serological and ultrasound parameters, so that only higher-risk patients undergo MRCP. Secondly, we recommend that IOC be performed more routinely where appropriate, allowing patients to have cholecystectomy and bile duct analysis as one procedure. The benefit of this is threefold: it relieves pressure on MRCP services (which are not always widely available), reduces pre-operative investigatory delays, and can shorten length of stay (46, 119). Stricter MRCP use has also been shown to reduce cost (120, 121).

Pre-operative ERCP for removal of CBD stones can cause similar delays. Laparoscopic bile duct exploration or intra-operative ERCP *alongside* laparoscopic cholecystectomy are both viable alternatives (43) that are equally as successful as pre-operative ERCP, with no significant difference in morbidity or mortality (45). As well as expediting cholecystectomy, this obviates an uncomfortable procedure, as patients will already be anesthetized for their cholecystectomy.

Another option would be to make pre-operative MRCP/ ERCP more available. Davies et al. (122) found that introducing a non-urgent upper GI endoscopy service at the weekend reduced length of inpatient stay without increased mortality. Resource leveling studies on ERCP have also proposed a half-day Saturday list (123).

Similarly, a “Surgeon of the week” model could be introduced, as trialed by Agrawal et al. (124). They rostered an on-call consultant 6-days a week, from 8 am to 8 pm, on a 10-week cycle. This resulted in more cholecystectomies being done on index admission, without increase in morbidity.

For surgeons with ongoing reservations on early cholecystectomy, further training could be provided to familiarize “hot” gallbladder operations. Mercer et al. (125), found that integrating a specialist upper gastrointestinal surgical team (with 2 full-time and 2 part-time consultants) led to more acute cholecystectomies (67.3% from 37.3%), fewer readmissions, generally lower conversion rates and shorter hospital stay.

A final suggestion would be to alter how we prioritize theater spaces. The story of patients' cholecystectomies being intended for index admission and then being canceled and postponed is all too common. One solution could be to have dedicated acute cholecystectomy theater lists, either in addition to or in lieu of some elective lists. Alternatively, a separate “urgent” operating theater could run alongside the CEPOD list, so that fewer patients' procedures are canceled for emergencies. A South-East Wales study calculated that to treat 787 cholecystitis patients on index admission, there would need to be 12 cholecystectomies per week, across 5.4 operating sessions (126). Whilst NHS resource availability may not allow for this many weekly sessions, even having two sessions per week would help. If a morning list was done on a Monday and a Thursday, then the maximum a patient would have to wait for an index procedure would be 96 h (presuming there were no additional opportunistic spaces on other theater lists).

## Discussion

There were a few discussion points for this review. Firstly, not all studies had the same definitions for “late” or “early” cholecystectomy. For instance, some studies specified “late” as >6 weeks post initial presentation, for others it was >7 days. “Early” meant <72 h for some researchers, <96 h for others, and <7 days for a remainder. There therefore was likely to be some crossover between “late” and “early” groups between some studies, although the unanimous finding remains that “early” is safer and cheaper, regardless of differences in time criteria.

Secondly, many of the studies discussed were conducted at large tertiary care centers. Cameron and Goodman (127) examined gallstone pancreatitis outcomes in a UK district general hospital (DGH) and deemed early laparoscopic cholecystectomy “safe and feasible” without increased surgical difficulty. However, others have discussed a potential lack of sufficiently trained surgeons for early cholecystectomies in the DGH setting (126).

A final point to note is that much of the literature is focused toward cholecystectomy in the context of acute cholecystitis. Further research into cholecystectomy for other biliary pathologies might be useful; initial papers do highlight promising results.

## Conclusion

Gallstone disease accounts for a large proportion of general surgical admissions. Early laparoscopic cholecystectomy has been shown to be safe with reduced rates of bile leak, morbidity and complications, shorter inpatient stay, and huge financial savings for hospital trusts and NHS as a whole. Performing the procedure early also greatly benefits the individual, with better quality of life, fewer days off work and reduced re-admission to hospital.

Services should change to reflect this, but obstacles such as imaging delays and changeable operating theater slots persist. We have proposed several ways to overcome these, but they require a systemic and NHS-wide change in approach. If achieved, significant improvements in patient care can be made.

## Author Contributions

The authors state equal contribution in the preparation of this manuscript. All authors were involved in writing the article and preparing for submission.

## Conflict of Interest

The authors declare that the research was conducted in the absence of any commercial or financial relationships that could be construed as a potential conflict of interest.
